# Indocyanine green plasma disappearance rate for assessment of liver function: re-evaluation of normal ranges and impact of biometric data

**DOI:** 10.1186/cc12116

**Published:** 2013-03-19

**Authors:** W Huber, M Kranzmayr, C Schultheiss, W Reindl, A Krug, B Saugel, U Mayr, RM Schmid

**Affiliations:** 1 Klinikum rechts der Isar, Technical University of Munich, Germany

## Introduction

The indocyanine green plasma disappearance rate (ICG-PDR) is a dynamic liver function test that can be non-invasively measured by pulse densitometry. ICG-PDR is associated with mortality and other markers of outcome. Due to predominant use of ICG-PDR in the ICU setting, the normal range is based on scarce data available outside the ICU and given with 18 to 25%/minute.

## Methods

To prospectively re-evaluate the normal range and to analyze the potential impact of biometric data on ICG-PDR, we measured ICG-PDR (i.v. injection of 0.25 mg/kg ICG; LiMON, Pulsion, Munich, Germany) in 95 outpatients with ulcerative colitis (UC). Due to a prevalence of primary sclerosing cholangitis (PSC) of 5% in UC, patients were asked regarding previous diagnosis of PSC. Additionally, serum bilirubin, AST, ALT, INR, AP, γGT and cholinesterase were determined. Association of biometric data (age, gender, height, weight) and diagnosis of PSC on ICG-PDR were evaluated using Spearman correlation, ROC and multivariate analysis (statistics: IBM SPSS 20).

## Results

A total of 95 patients (54 male, 41 female), age 43.2 ± 15.5 (21 to 76) years, weight 74 ± 16 kg, height 175 ± 10 cm, BMI 23.7 ± 4, bilirubin 0.48 ± 0.27 mg/dl, AST 26.8 ± 11.4 U/l, ALT 26.8 ± 14.9 U/l, previous diagnosis of PSC 8/95 (8.4%). ICG-PDR ranged from 13.8 to 44.0 with a mean of 28.2 ± 6.8 and a median of 27.0%/minute. In univariate analysis ICG-PDR was significantly associated with age (*r *= -0.480; *P <*0.001), weight (*r *= -0.262; *P *= 0.011) and female gender (*r *= 0.221; *P *= 0.032), but not to height (*P *= 0.681). In multivariate analysis (*R *= 0.459) including the variables age, gender, height, weight and diagnosis of PSC, only age was independently (*P <*0.001) associated with ICG-PDR. With each year in age, ICG-PDR decreased by 0.206%/minute. In ROC analysis, ICG-PDR above the upper normal range (>25%/minute) was significantly associated with young age (AUC 0.746; *P <*0.001).

## Conclusion

ICG-PDR (normal) values should be corrected for age. With a decrease in ICG-PDR of 0.206% per year, the range between the upper and lower normal level (25 to 18%/minute) is passed through within about 35 years of life. These findings are in accordance with functional loss of other organ functions (for example, cardiac output, glomerular filtration rate) with increasing age. Normal ranges of innovative markers of organ function mainly derived from ICU populations should be re-evaluated outside the ICU.

